# Ictal eructation in a case of idiopathic generalized epilepsy

**DOI:** 10.1016/j.ebr.2025.100820

**Published:** 2025-08-09

**Authors:** Amirtha Shekar, Sreekanth Koneru, Charles Ákos Szabó

**Affiliations:** a Long School of Medicine, UT Health San Antonio, 7703 Floyd Curl Dr, San Antonio, TX 78229, USA; b Department of Neurology, UT Health San Antonio, 7703 Floyd Curl Dr, San Antonio, TX 78229, USA; c South Texas Comprehensive Epilepsy Center, 7703 Floyd Curl Dr, San Antonio, TX 78229, USA

**Keywords:** Ictal eructation, Idiopathic generalized epilepsy, Epilepsy monitoring unit, Seizure semiology

## Abstract

We present a 57 year-old right-handed female with a history of idiopathic generalized epilepsy since age two years old as well as mild-to-moderate intellectual impairment and behavioral dyscontrol. She was seizure free for many years on ethosuximide, but her absence seizures recurred as her dose was gradually decreased. After raising her dose, she also began to experience falls, at times with decreased responsiveness. She was admitted for inpatient video-EEG monitoring for quantification of her absence seizures and characterization of her falls. Our patient had 3–6 absence seizures per hour. In addition to a brief alteration of awareness, her absence seizures were frequently associated with belching and eyelid myoclonia lasting for 3–4 s. Her belching was correlated with a generalized ictal discharge and was not noted interictally. Several episodes of unsteadiness were noted, at times the patient falling backwards into her bed, but never falling to the ground from a standing position; none of these episodes associated with ictal EEG correlate. In summary, our patient demonstrated and absence seizures with ictal eructation or belching, which has not been reported as an ictal symptom of generalized seizures, and episodes of unsteadiness of unknown characterization.

## Highlights

• Ictal eructation has been observed in focal, but not generalized epilepsy.
• On EEG, eructation during absence seizures in our patient shows 3–6 Hz discharges.
• Ethosuximide dose increase was shown to resolve eructation in our patient.
• The insular network and mesio-temporal lobe are postulated to elicit eructation.

## 1 Introduction

Eructation, or belching, is a rare ictal symptom described in only two cases so far [1,2]. The first case was described in a letter to the editor with minimal semiological detail [1]. These episodes were not associated with loss of awareness, and during one episode “the EEG showed paroxysmal 4 Hz activity…*not* confined to the left hemisphere but was more prominent in the left temporal area”. The second case report demonstrated that ictal eructation was part of a focal impaired awareness seizure occurring seconds before evolution into a bilateral tonic-clonic seizure; the ictal discharge was characterized by a generalized attenuation eventually evolving into a right frontotemporal discharge [2]. In addition to a single video-EEG recorded case of focal epilepsy, eructation has also been described in association with psychogenic non-epileptic semiologies [3], but not in the context of an idiopathic generalized epilepsy (IGE) [3].

This clinical vignette describes a 57 year-old right-handed woman with absence seizures with eyelid myoclonia presenting with ictal eructation. She was admitted to the epilepsy monitoring unit (EMU) at University Hospital in San Antonio due to an increased frequency of absence seizures and falls of unclear characterization. Eructation was noted frequently in the setting of absence seizures associated with eyelid myoclonia and correlated with a generalized ictal discharge.

## 2 Case study

Our patient had a history of absence seizures since age 2 years old as well as mild-to- moderate intellectual disability of unknown etiology, anxiety and behavioral dyscontrol. Her mother had a history of myoclonic seizures in her teenage years. The patient’s family provided a history of generalized stiffening episodes with falls as a child, which became well-controlled with valproate and ethosuximide (ESM). Later, at age 40 years old, due to side-effects from valproic acid, which included fatigue and a postural tremor, she was converted to ESM monotherapy for the treatment of absence seizures without recurrence of generalized stiffening episodes. She had generalized 3–5 Hz spike-and-wave discharges recorded on routine outpatient EEGs as well as posterior predominant photoparoxysmal responses. Brain MRI scans were always normal, including 3 T MRI scans with and without gadolinium utilizing seizure protocols. In her sixth decade, her absence seizures appeared to have remitted, and her outpatient routine EEG demonstrated a normal awake and sleep background; her ESM was gradually weaned off as she complained of fatigue on the medication. However, within a few weeks, her absence seizures recurred, requiring resumption of EMS at a lower dose 250 mg daily, which was not associated with any side effects. Apart from her absence seizures, she was also noted to suffer from falls. Eventually, clonazepam 1 mg was also added, in part for seizure control and in part to treat her anxiety and insomnia.

**Fig. 1:**
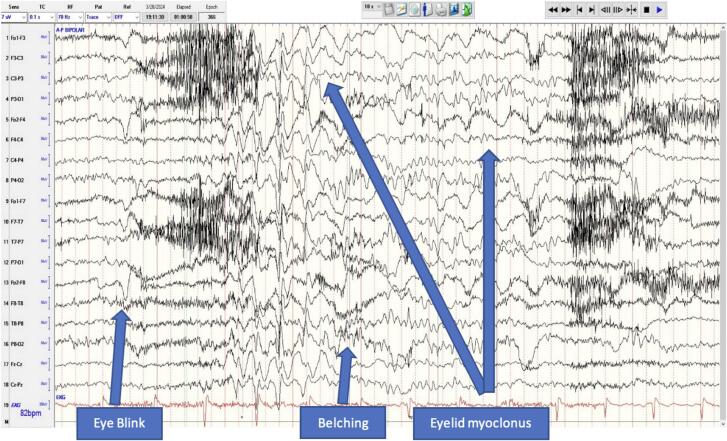
Scalp EEG Recording of an Eyelid Myoclonus Associated with an Ictal Eructation. EEG performed at the EMU for a 4-day period. AP bipolar recording captures a typical absence seizure beginning with a 3–5 Hz spike-and-wave discharge followed by eructation and eyelid myoclonus. Note the absence of muscle artifact during the ictal discharge.

The patient was admitted for four days to the Epilepsy Monitoring unit (EMU) at University Hospital in San Antonio, Texas, to quantify her burden of absence seizures and characterize her falling spells. She maintained on her ESM during her hospitalization. She had frequent, subtle eyelid myoclonia with absence seizures 3–6 times per hour while awake, all of which lasted 4–8 s. She exhibited eructation in about 40 % of seizures. The ictal discharges were characterized by a 3–5 Hz generalized paroxysmal spiking followed by eyelid myoclonia during which ictal eructation was noted (Fig. 1). When her ethosuximide dosage was increased to 500 mg twice daily, her absence seizures decreased and the ictal eructation resolved, without immediate complaints of fatigue.

While we did not record any episodes of eructation without an EEG correlate while she was in the EMU, her sister did report that she has a history of belching; the belching was not well-correlated to meals, and tended to occur randomly, at times even appearing as an attention-seeking behavior. She had a previous history of a hemicolectomy for a low grade papillary mucinous appendiceal neoplasm and was diagnosed with gastroesophageal reflux disease (GERD) without esophagitis by endoscopy.

Some of her episodes of unsteadiness and falling were recorded but none were associated with an EEG correlate. She appeared to have momentary unsteadiness when standing up from the bed or the toilet, at times falling backwards into her bed when she was sitting on the bed or just about to stand up. While experiencing the unsteadiness while standing, she would sway and then tighten her grip on her walker, avoiding the fall. One on or two occasions it appeared that one of her lower extremities was about to buckle. Orthostatic hypotension was occasionally documented during these episodes outside of the hospital. She had no cardiac arrhythmias and a cardiac work-up for syncope was also negative. A carotid duplex study demonstrated asymptomatic carotid stenoses < 50 % bilaterally.

## 3 Discussion

Eructation is an extremely rare ictal symptom. This is only the third case report of ictal eructation and only case associated with video-EEG documented generalized epilepsy. During our patient’s absence seizures, eructation tended to follow the initial generalized spike-and-wave discharge with a variable latency but always occurring during the eyelid myoclonia. Eructation in this patient may have been in part due to her history of GERD as well as excessive supragastric belching, a functional condition which can be activated semi-voluntarily through suggestion, similar to psychogenic nonepileptic episodes [3,4]. As she was unaware of her eructation ictally, it was unlikely to be driven by suggestible or semi-voluntary behaviors. It was described as an automatism in one of the previously reported two cases, as it was repetitive and occurred in the setting of impaired awareness [2].

On a physiological level, eructation is a reflex initiated in response to an excess volume of air in the stomach [5]. The accumulation of air stretches the gastric wall, thus activating receptors and relaxing the lower esophageal sphincter allowing the air to rush upwards and be expelled as a belch. The area postrema (AP) is the subcortical structure responsible for mediating eructation, with studies demonstrating that lesions to the AP inhibit eructation. While abdomino-visceral symptoms have been activated by electrocortical stimulation in stereotactic or intracranial grid and strip electrode studies, eructation was never reported [6,7]. In the other two cases with electroclinical documentation, ictal eructation occurred in the setting of a diffuse bi-hemispheric ictal discharge; our case further confirms that a broader cortical ictal deactivation may lead to the activation or disinhibition of particular brainstem-mediated reflexes, mechanisms that also have been suggested to underlie ictal emesis [6,7].

Privacy rights of patient were followed and the authors obtained informed consent from the patient prior to utilization of patient data. Study was performed in compliance with relevant laws and institutional guidelines.

## CRediT authorship contribution statement

**Amirtha Shekar:** Writing – review & editing, Writing – original draft, Visualization, Investigation, Data curation, Conceptualization. **Sreekanth Koneru:** Writing – review & editing, Validation, Supervision, Software, Resources, Project administration, Methodology, Data curation, Conceptualization. **Charles Ákos Szabó:** Writing – review & editing, Validation, Supervision, Project administration.

## Declaration of competing interest

The authors declare the following financial interests/personal relationships which may be considered as potential competing interests: CAS and SK are participating in the XENON XTOLE 301 & 304, and AS does not have any conflicts of interest to disclose.
